# Biomass- and Carbon Dioxide-Derived Polyurethane Networks for Thermal Interface Material Applications

**DOI:** 10.3390/polym16020177

**Published:** 2024-01-07

**Authors:** Ji Won Jang, Inhwan Cha, Junhyeon Choi, Jungwoo Han, Joon Young Hwang, Il Gyu Cho, Seung Uk Son, Eun Joo Kang, Changsik Song

**Affiliations:** 1 Department of Chemistry, Sungkyunkwan University, Suwon 16419, Republic of Korea; jiwon10236@g.skku.edu (J.W.J.); ddrfgt@hotmail.com (I.C.); hjw4577@g.skku.edu (J.H.); 379cho35@gmail.com (I.G.C.); sson@iskku.edu (S.U.S.); 2 Department of Applied Chemistry, Kyung Hee University, Yongin 17104, Republic of Korea; chk1800613@naver.com (J.C.); jyhwang08@khu.ac.kr (J.Y.H.)

**Keywords:** CO_2_ utilization, biomass-derived monomer, dynamic covalent bond, mechanochemical synthesis, thermal interface material

## Abstract

Recent environmental concerns have increased demand for renewable polymers and sustainable green resource usage, such as biomass-derived components and carbon dioxide (CO_2_). Herein, we present crosslinked polyurethanes (CPUs) fabricated from CO_2_- and biomass-derived monomers via a facile solvent-free ball milling process. Furan-containing bis(cyclic carbonate)s were synthesized through CO_2_ fixation and further transformed to tetraols, denoted FCTs, by aminolysis and utilized in CPU synthesis. Highly dispersed polyurethane-based hybrid composites (CPU–Ag) were also manufactured using a similar ball milling process. Due to the malleability of the CPU matrix, enabled by transcarbamoylation (dynamic covalent chemistry), CPU-based composites are expected to present very low interfacial thermal resistance between the heat sink and heat source. The characteristics of the dynamic covalent bond (i.e., urethane exchange reaction) were confirmed by the results of dynamic mechanical thermal analysis and stress relaxation analysis. Importantly, the high thermal conductivity of the CPU-based hybrid material was confirmed using laser flash analysis (up to 51.1 W/m·K). Our mechanochemical approach enables the facile preparation of sustainable polymers and hybrid composites for functional application.

## 1. Introduction

Petroleum-based plastics have been widely used since they were first developed in 1907 [1] because plastics offer many advantages, including the fact that they are lightweight, inexpensive to produce, and their properties can be adjusted as desired [2]. For example, the production of polyurethane has gradually increased over time [3]. This is because, using a combination of monomers, the physical properties of polyurethane can be freely adjusted to adapt it to specific purposes. Furthermore, polyurethane acquires durability through the hydrogen bonding of urethane moieties and, thus, it is used for various purposes, from soft adhesives to special-purpose materials [4,5]. However, because most polymers, including polyurethanes, are discarded in landfills or are incinerated after use, they cause serious environmental pollution [6,7,8,9]. Therefore, recycling polymers has been an active area of research with growing importance. In principle, thermoplastics can be recycled because they become malleable when they are heated [10]. However, thermosets have excellent chemical resistance and durability but are difficult to recycle because they have crosslinked structures. Therefore, efficient recycling methods are needed for thermosetting polymers [11].

One effort to achieve efficient recycling is the fabrication of crosslinked polymers with dynamic covalent bonds (DCBs) in the polymer chain, called covalent adaptable networks (CANs) [12,13,14]. A DCB refers to a chemical bond that can form and break under certain conditions, such as heat, light, pH, etc. Some representative chemical reactions using DCBs include Diels–Alder, disulfide exchange, boronic ester exchange, and urethane exchange reactions (transcarbamoylation) [15]. Given the reversible nature of DCBs, CANs are reconfigurable, self-healable, and reusable [16]. Since Leibler first reported epoxy polymers with an exchangeable β-hydroxy ester (coined “vitrimer”) in 2011 [17], various CANs have been developed actively [18,19,20,21]. CANs may be a solution for developing environmentally friendly, recyclable, and sustainable polymers, including thermosets.

Meanwhile, to achieve carbon neutrality, making recyclable materials and using resources derived from biomass or CO_2_ capture and utilization is important [22,23,24]. Furthermore, developing synthesis methods that minimize production waste materials, such as solvents and byproducts, is necessary [25]. The CO_2_ incorporation into polymers is generally conducted by direct copolymerization with epoxides or indirect polymerization through ring-opening in cyclic carbonates [26]. However, lignocellulose-derived platform chemicals show promise as biomass-derived materials for polymer synthesis as they can be transformed into monomers or other building blocks for existing and novel polymers [27]. Among lignocellulose-derived platform chemicals, aliphatic acids and alcohols, such as succinic acid, lactic acid, itaconic acid, isosorbide, and 1,4-butanediol, are widely utilized in polymer synthesis [28]. In addition, aromatic platform chemicals, such as vanillin, diphenolic acid, and 2,5-furandicarboxylic acid, are utilized in polymer synthesis [29]. However, 2,5-bis(hydroxymethyl)furan (BHMF), a lignocellulosic furan derivative, has been less frequently used, although it can be obtained easily from various biomasses, such as waste wood [30]. Our group has previously reported that thermoplastic or thermoset polymers can easily be prepared using BHMF via a facile, solvent-free ball milling process and can be used for self-healable, reprocessable, and shape-memory materials [31,32]. However, synthetic strategies involving biomass- and CO_2_-derived monomers are still quite rare, despite the increasing number of reports on sustainable polymers fabricated from each renewable source [33].

The development of electronic devices enables a convenient lifestyle, but overheating of the heat source (chip) due to high integration of electronic devices can cause electronic failure, which can be quite dangerous. Therefore, developing materials that dissipate heat properly is necessary [34]. For heat to dissipate, a heat sink is installed on the electronic device heat source, but the rough interface between the two components hinders heat transfer because of the air gap it creates [35,36]. Thus, thermal interface materials (TIMs) have been developed to reduce the interface thermal resistance between the heat sink and heat source [37]. Some examples of these materials are thermal grease, gel, and tape, but most of the materials are developed as composites of a polymer matrix and thermal conductive fillers [38]. This strategy is designed to compensate for the limitations of inorganic conductive fillers and polymer materials: inorganic conductive fillers have high thermal conductivity but poor adhesion, whereas polymer materials have excellent adhesion but low thermal conductivity [39,40,41]. Epoxy, silicon, and thermoplastic polyurethane are typical polymers used to develop TIMs [39]. Of these, polyurethanes have high water resistance and adjustable hardness and elasticity [42,43]. Furthermore, urethane bonds may enhance the adhesion between fillers and polymer [44], and they provide stress relief when used in composite interfaces [45]. Typically, thermoset polymers are difficult to recycle but preferred over thermoplastic polymers because their high crosslink density can enhance the thermal conductivity (κ) of TIMs [46]. However, their matrix stiffened with thermoset polymer may render conformal contact difficult, resulting in high contact resistance (R_c_) [47]. Recently, we reported that DCB played an important role in lowering R_c_ in a thermoset polymer-based TIM because of its ability to conform at the contact interface [47].

Herein, we introduce BHMF-based network polyurethanes (crosslinked polyurethanes, CPUs) fabricated through mechanochemical polymerization to have a dynamic covalent chemistry, and we analyze their thermophysical and rheological properties. Particularly, we develop crosslinkers that capture CO_2_ in the biomass-derived BHMF. The properties of exchangeable DCBs in the network polymers, which give malleability to the network polyurethanes, are confirmed through our rheological analysis. Finally, a composite of the sustainable polyurethane and a thermal conductive filler (Ag) is developed, and its ability to transfer heat is validated. We demonstrate that novel biomass-derived network polyurethanes that directly utilize biomass and CO_2_ can be prepared using a facile ball milling process and successfully used in composite materials that serve as TIMs.

## 2. Materials and Methods

### 2.1. Materials

All chemicals were commercially available, except CO_2_-captured furan carbamate tetraols (FCTs), and they were used as received without further purification, except for BHMF. The 1,6-hexanediol (>97%), dibutyltin dilaurate (DBTDL, >95%), and hexamethylene di-isocyanate (HDI, >98%) were purchased from TCI (Tokyo, Japan). Silver (Ag) flakes were purchased from Metalor (Metalor, SA-31812, Neuchatel, Switzerland). BHMF was purchased from Nanjing Sunshine Chemical Co., Ltd. (Nanjing, China), and was used after recrystallization.

### 2.2. Synthesis of CO_2_-Captured FCT

#### 2.2.1. General Procedure for 2,5-bis((oxiran-2-ylmethoxy)methyl)furan (BOMF) Synthesis

Epichlorohydrin (55.0 mL, 0.70 mol, 14.0 equiv.) was poured into a two-necked round bottomed flask. A 50.0 wt% solution of NaOH (24.0 g, 0.60 mol, 12.0 equiv.) was added, and n-Bu_4_NHSO_4_ (1.70 g, 5.00 mmol, 0.10 equiv.) was used as a transfer catalyst. The solution was stirred for 30 min at 25 °C under an argon atmosphere. A solution of BHMF (6.41 g, 0.05 mol, 1.00 equiv.) in tetrahydrofuran (THF) (40.0 mL) was added dropwise to the reaction, and the reaction mixture was heated to 60 °C. After the mixture was stirred for 4 h, the reaction was stopped by adding ice water to the mixture. The reaction mixture was extracted with ethyl acetate (EtOAc) and dried over MgSO_4_, and the solvent was then removed under reduced pressure to obtain a crude mixture. The crude mixture was purified by flash column chromatography to obtain 2,5-bis((oxiran-2-ylmethoxy)methyl)furan (BOMF) as a viscous brownish oil (11.0 g, 91.5%); R_f_ = 0.18 (hexane/EtOAc, 2:1); ^1^H NMR (400 MHz, CDCl_3_) δ 6.29 (s, 2H), 4.55−4.46 (m, 4H), 3.77 and 3.43 (ABX, J_AB_ = 11.7 Hz, J_AX_ = 3.2 Hz, J_BX_ = 5.8 Hz, 4H), 3.17 (ddt, J = 5.8, 3.5, 2.7 Hz, 2H), 2.81 and 2.62 (ABX, J_AB_ = 6.9 Hz, J_AX_ = 4.0 Hz, J_BX_ = 2.6 Hz, 4H) ppm.

#### 2.2.2. General Procedure for 4,4’-(((furan-2,5-diylbis(methylene))bis(oxy)) bis(methylene))bis(1,3-dioxolan-2-one) Synthesis

BOMF (5.29 g, 22.0 mmol, 1.00 equiv.), n-Bu_4_NBr (0.355 g, 1.10 mmol, 0.05 equiv.), and CH_2_Cl_2_ (17.7 mL) were placed in a 50 mL stainless-steel reactor. The reaction was performed under 10 bar CO_2_ pressure, and the mixture was stirred at 80 °C for 24 h. The conversion of epoxide to cyclic carbonate was then determined by analyzing a sample using ^1^H NMR spectroscopy. The crude reaction sample was filtered through a pad of silica and eluted with CH_2_Cl_2_ to remove the catalyst. The solvent was then evaporated in a vacuum to render the crude cyclic carbonate product, purified through recrystallization by adding MeOH to produce the 4,4′-(((furan-2,5-diylbis(methylene))bis(oxy))bis(methylene))bis(1,3-dioxolan-2-one) (5.78 g, 80.0%); R_f_ = 0.12 (hexane/EtOAc, 1:2); ^1^H NMR(400 MHz, CDCl_3_) δ 6.31 (s, 2H), 4.79 (m, 2H), 4.49 and 4.38 (ABX, J_AB_ = 12.1 Hz, J_AX_ = 7.4 Hz, J_BX_ = 6.0 Hz, 8H), 3.74 and 3.65 (ABX, J_AB_ = 11.1 Hz, J_AX_ = 3.2 Hz, J_BX_ = 3.6 Hz, 4H) ppm.

#### 2.2.3. General Procedure for FCT Synthesis

A mixture of 4,4′-(((furan-2,5-diylbis(methylene))bis(oxy))bis(methylene))bis(1,3-dioxolan-2-one) (1.0 equiv.) and amino alcohol (5.0 equiv.) in THF was stirred at room temperature under an argon atmosphere for 12 h. After the solvent was removed under reduced pressure, the crude mixture was purified using flash column chromatography (CH_2_Cl_2_/MeOH).

FCT-1: Cyclic carbonate (0.28 g, 0.87 mmol, 1.0 equiv.) and 2-amino ethanol (0.26 mL, 4.3 mmol, 5.0 equiv.) were treated using THF (8.7 mL), and the crude mixture was purified according to the general procedure (CH_2_Cl_2_/MeOH, 15:1). Purified FCT-1 was obtained as a yellowish liquid (0.30 g, 77%). R_f_ = 0.12 (CH_2_Cl_2_/MeOH, 9:1), ^1^H NMR (400 MHz, CD_3_OD) δ 6.31 (s, 2H), 4.43 (s, 4H), 4.07–3.79 (m, 6H), 3.58–3.40 (m, 8H), 3.19–3.15 (m, 4H) ppm, and HRMS (ESI): calculated for C_18_H_30_N_2_O_11_Na [M+Na]^+^ 473.1742, found 473.1745.

FCT-2: Cyclic carbonate (1.3 g, 4.0 mmol, 1.0 equiv.) and 2-amino-1-phenylethanol (2.7 g, 20. mmol, 5.0 equiv.) were treated using THF (40 mL), and the crude mixture was purified according to the general procedure (CH_2_Cl_2_/MeOH, 20:1). The purified FCT-2 obtained was a whitish liquid (1.30 g, 54.0%). R_f_ = 0.19 (CH_2_Cl_2_/MeOH, 9:1), ^1^H NMR (400 MHz, CD_3_OD) δ 7.37–7.32 (m, 6H), 7.31–7.22 (m, 4H), 6.33 (s, 2H), 4.72–4.69 (t, 2H), 4.46 (s, 4H), 4.09–3.84 (m, 6H), 3.63–3.23 (m, 8H) ppm, and HRMS (FAB): calculated for C_30_H_38_N_2_O_11_ [M+H]^+^ 603.2548, found 603.2548.

FCT-3:A mixture of carbonate (0.36 g, 1.1 mmol, 1.0 equiv.) and 2-amino-3-methyl-1-butanol (0.61 mL, 5.5 mmol, 5.0 equiv.) in THF (11 mL) was charged with argon, and the reaction mixture was stirred at 50 °C for 5 days. After it was cooled to room temperature, the crude mixture was purified according to the general procedure (CH_2_Cl_2_: MeOH, 40:1). The purified FCT-3 obtained was a yellowish liquid (0.24 g, 40.0%). R_f_ = 0.80 (CH_2_Cl_2_/MeOH, 4:1), ^1^H NMR (400 MHz, CD_3_OD) δ 6.33 (s, 2H), 4.46 (s, 4H), 4.11–3.86 (m, 6 H), 3.32–2.95 (m, 10H), 1.88–1.78 (m, 2H), 0.97–0.87 (m, 12H) ppm, and HRMS (FAB): calculated for C_24_H_42_N_2_O_11_ [M+H]^+^ 535.2867, found 535.2863.

### 2.3. Synthesis of Ag-Nanoparticle-Coated Multiwalled Carbon Nanotubes (nAgMWNTs)

AgNO_3_ in ethanol (Kojundo Chemical, Sakado, Japan, 0.02 mol L^−1^, 400 mL) and benzyl mercaptan in ethanol (Sigma Aldrich, St. Louis, MO, USA, B25401, 0.1 mol L^−1^, 3.2 mL) were stirred for 48 h to synthesize a phenyl group-functionalized Ag-nanoparticle solution. MWNTs dispersed in ethanol (Timesnano, Chengdu, China, TNGM2, outer diameter, 8–15 nm; length, 10–15 μm; 133 mg, 200 mL) were added to the Ag-nanoparticle solution and then sonicated (Lab Companion, Daejeon, Republic of Korea, UCP20, 3 h). Finally, the nAgMWNTs were synthesized using vacuum filtration and were dried in a vacuum desiccator at 25 °C for 24 h.

### 2.4. Network Polyurethane (CPU) Using CO_2_

CO_2_-utilizing network polyurethane (CPU) was synthesized using the ball milling method. BHMF (61 mg, 0.47 mmol, 1 equiv.), 1,6-hexanediol (56 mg, 0.47 mmol, 1 equiv.), FCTs (approximately 200–340 mg, 0.47 mmol; 2 equiv.), DBTDL (1 mol%), and HDI (218 mg, 1.42 mmol; 6 equiv.) were added to a 25 mL stainless-steel vessel along with two stainless-steel balls (15 mm diameter). The stainless-steel vessel containers were mixed on a mixer at a vibration frequency of 20 Hz for 60 min at room temperature. The reacted solid polymer was collected without any further purification. FT-IR (cm^−1^): 3325 (N–H stretching), 1710 (NH–CO–O stretching; urethane moiety).

### 2.5. Preparation of CPU–Ag Composites (CPU-TIM) Using Ball Milling Method

CPU-TIM was synthesized using the ball milling method. BHMF (61 mg, 0.47 mmol, 1 equiv.), 1,6-hexanediol (56 mg, 0.47 mmol, 1 equiv.), FCTs (approximately 200–340 mg, 0.47 mmol; 2 equiv.), DBTDL (1 mol%), HDI (218 mg, 1.42 mmol; 6 equiv.), Ag flakes (29.5 vol% weight of polymer portion), and nAgMWNTs (0.5 vol% weight of polymer portion) were added to a 25 mL stainless-steel vessel along with two stainless-steel balls (15 mm diameter). The stainless-steel containers were mounted on a mixer and mixed at a vibrational frequency of 20 Hz for 60 min at room temperature. The reacted solid polymer was collected without any further purification. FT-IR (cm^−1^): 3325 (N–H stretching), 1710 (NH–CO–O stretching; urethane moiety).

### 2.6. Preparation of CPU and CPU-TIM Film Using Hot Press

Synthesized CPU, or CPU-TIM powder, was uniformly spread over a 0.5 mm thick stainless-steel mold and then hot-pressed using a KP-2200 hot press at 130 °C for 2 h at 30 MPa pressure. The prepared film was then cured with the press at 160 °C for 4 h at 30 MPa pressure.

### 2.7. Characterization Methods

Fourier transform infrared (FT-IR) spectroscopy was performed using a Bruker Optics VERTEX 70 (Billerica, MA, USA) spectrometer. The spectra were recorded using a diamond-attenuated total reflection unit in the spectral range of 4000–600 cm^–1^.

Dynamic mechanical thermal analysis (DMTA) and stress relaxation analysis (SRA) were performed using an Anton-Paar MCR 302e (Graz, Austria) in an air environment. The dimensions of the sample were 8 × 8 × 0.5 mm^3^. The samples were heated to the desired isothermal temperature. Measurements of the storage modulus (G′), loss modulus (G″), and tan delta (δ) were recorded as functions of temperature as the sample was heated from −30 °C to 180 °C at a 3 °C min^−1^ heating rate. The measurement for DMTA was performed at 1 Hz frequency and 0.1% strain. The measurement for SRA was taken in shear mode as a constant 1% strain was applied to the sample. A constant normal force of 5 N was applied throughout the measurement to ensure good contact between the material and the instrument. The sample was allowed to equilibrate at a particular temperature (140 °C, 150 °C, 160 °C) for 10 min before applying the strain.

Differential scanning calorimetry (DSC) was performed using a DSC 7020 (Hitachi High-Tech, Tokyo, Japan) in N2 condition. The sample was prepared as a powder. The glass transition temperature was measured as a function of temperature over a range from room temperature to 150 °C at a 10 °C/min heating rate.

Thermogravimetric analysis (TGA; Seiko Exstar 6000 (TG/DTA6100, Chiba, Japan)) was carried out in the air. The sample weight was approximately 10 mg. The sample was measured over a range from room temperature to 600 °C at a 10 °C/min heating rate.

Laser flash analysis (LFA) was performed using a LFA 467 (Netzsch, Selb, Germany) to measure thermal diffusivity (α). The density (ρ) of specimens was measured according to the Archimedes method (Sartorious, Gottingen, Germany, Quintix 224-1 SKR). Finally, the thermal conductivity (κ) was obtained using κ = Cp α ρ.

## 3. Results

### 3.1. Solid-State Synthesis of CO_2_-Utilizing Network Polyurethanes (CPUs) Using a Biomass-Derived Alcohol and CO_2_ Fixation Crosslinker

**Scheme 1:**
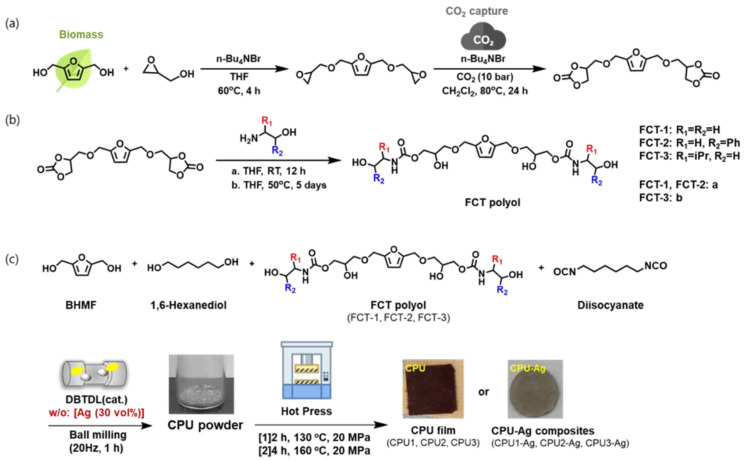
(**a**–**c**) CO_2_-utilizing network polyurethanes (CPUs) and the film with CPU and thermal filler composites (CPU–Ag) via ball milling and the hot-press method.

Previously, our group reported thermoplastic and malleable thermoset polyurethane synthesis using BHMF, di-isocyanate, and a DBTDL catalyst via mechanochemical polymerization [31,32]. The ball milling synthesis is an eco-friendly and energy-saving method, proving that polyurethane formation can be successfully performed by supplying friction energy with controlled frequency and reaction time at room temperature in a short time. During the synthesizing process of network-type polyurethane, crosslinkers are essential to form a network structure as they interconnect the polymer chains. Typically, multifunctional substances having at least three functionalities are used as the crosslinker. In this study, FCTs were synthesized by CO_2_ utilization on BHMF and as a crosslinking agent in the preparation of CPUs by the ball milling method (Scheme 1).

CPUs were successfully obtained as a gray powder synthesized under the conditions of 20 Hz, 60 min, and vibrating ball milling with two stainless-steel balls (15 mm diameter). CPUs were composited with BHMF:1,6-hexandiol:FCT:diisocyanate at a 1:1:2:6 ratio, maintaining a stoichiometric balance between alcohol (OH) and isocyanate groups (NCO). Here, we used two diols, BHMF and 1,6-hexanediol, and the use of two types of diols in equal proportions was a conclusion reached during the optimization process in our previous studies with BHMF [3fp3]. This combination is to ensure that the synthesized CPUs have appropriate thermal and mechanical properties. If BHMF was used only as a diol, the results were not suitable because it produced rather brittle CPUs. Inclusion of 1,6-hexanediol as a flexible ingredient helped to enhance the mechanical properties of CPUs. In our previous studies, CPUs with lesser BHMF (vs hexanediol) resulted in decreased tensile strength due to the decreased content of the aromatic furan ring. Thus, we chose BHMF and 1,6-hexanediol in the same ratio (1:1) for the optimized conditions for CPUs. In addition, to utilize carbon dioxide as a valuable feedstock, we aimed to maximize the amount of FCTs since the carbon dioxide content was dictated by it. However, more than one equivalent of FCT with respect to the BHMF content resulted in CPUs with poor mechanical properties. Thus, in order to synthesize CPUs with appropriate physical and mechanical properties, the ratios were optimized to be 1:1:2:6 (BHMF: hexanediol: FCT-polyol: diisocyanate).

Additionally, to carry out the rheological analysis, films were prepared from the powder at an appropriate temperature and pressurized using hot-press equipment. Specifically, film preparation was carried out using a two-step process. First, in a precuring step, 30 MPa of pressure was applied at 130 °C for 2 h, and in a second step, a 4 h curing step was carried out under 30 MPa at 160 °C and without purification. In addition, CPU synthesis was confirmed by Fourier transform infrared spectroscopy (FT-IR) by comparing the CPU spectrum with starting materials, including 1,6-hexanediol, BHMF, DBTDL, HDI, and FCTs (Figure S1). In the FT-IR spectrum, the characteristic vibrations of the urethane bonds, carbonyl stretching at 1700 cm^−1^, and NH bonds at 3350 cm^−1^ were present in the network polyurethanes fabricated from FCTs, and the NCO peak for di-isocyanate had disappeared. This spectrum indicated that the urethane bonds had formed successfully during the ball milling and hot-press processes. The formation of network structures in CPU and CPU–Ag films was confirmed by a swelling test. After immersion in N,N′-dimethylformamide (DMF) for 24 h, the gel fraction of CPU was measured to be 73~84%, and the gel fraction of CPU–Ag was measured to be 94~99% which indicated the formation of well-crosslinked structures.

### 3.2. Thermal and Thermomechanical Characterization of CPU Films

**Figure 1:**
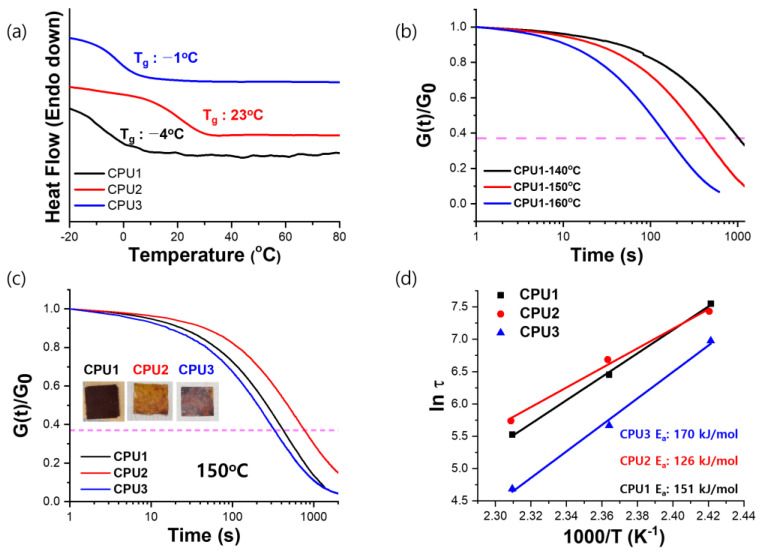
(**a**) DSC curves (2nd heating) of CPU1 (black), CPU2 (red), and CPU3 (blue); (**b**) normalized stress relaxation curves for CPU1 at three isothermal temperatures as a function of time; (**c**) comparison of normalized stress relaxation curves for CPU1 (black), CPU2 (red), and CPU3 (blue) at 150 °C; (**d**) Arrhenius plots of measured relaxation times for CPU1 (black), CPU2 (red), and CPU3 (blue).

We analyzed the thermal properties of CPU1, CPU2, and CPU3 using DSC and TGA. As shown by DSC profiles (Figure 1a), the glass transition temperatures (T_g_) of the film-type CPU1 and CPU3 were observed to be −4 °C and −1 °C. However, the T_g_ of the film-type CPU2 was observed to be much higher (23 °C), explained by the effect of FCT structures. When comparing the structural aspects of CPU1, CPU2, and CPU3, the main difference is whether the group (R_1_, R_2_, or R_3_) in the FCT polyol, which is the source of CPU, is an alkyl group or a phenyl group. When aromatic phenyl groups are introduced into polymers, they render the polymer more torsionally rigid and restrict the movement of the polymer chains. Therefore, CPU2, which is derived from FCT-2 with a phenyl group, has a much higher T_g_ than CPU1 or CPU3 (Figure 1a). Additionally, CPU3 has a slightly higher T_g_ than CPU1, because FCT-3, the source of CPU3, contains an isopropyl group, whereas FCT-1 has hydrogen, the smallest atom. The branched isopropyl may cause a torsional strain around the polymer chain, which can raise the T_g_.

Interestingly, we observed a higher T_g_ in the CPU “powder” compared to the CPU “film”. In fact, we anticipated that the film would exhibit a higher T_g_ due to more linkages. However, we observed a different outcome; the CPU powders have the T_g_s of 17~48 °C (Figure S2), while the films have values of −1~23 °C (Figure 1a). While we cannot provide an exact explanation, we considered a plausible rationale for this observation. Powder consists of smaller particles, possessing a larger surface area, allowing molecules greater freedom of movement, which may result in better intermolecular packings of oligomeric molecules. These packings require more energy to alter their states, raising the T_g_. On the other hand, when processed into films, more formation of urethane linkages happened, causing disrupted molecular packings. Consequently, we speculate that the film’s T_g_ might be lower than that of the powder.

Moreover, the decomposition temperatures at 5% weight loss (T_d_,5%) of the film-type CPU1 and CPU3 were below 200 °C (184 °C and 176 °C, respectively), whereas the T_d_,5% of film-type CPU2 was high (224 °C). This difference was interpreted as being caused by the phenyl group derived from FCT-2 (Figure S2).

To analyze the covalently adaptive networks of CPUs, SRA was performed on CPU films (CPU1, CPU2, and CPU3) under 140 °C–160 °C isothermal temperature conditions. In rheological studies of polymers, the determination of the small amplitude oscillatory shear (SAOS) area is very important. In the case of a large amplitude oscillatory shear (LAOS) region, it is not suitable to analyze dynamic covalent bonds because the deformation as well as viscoelastic properties of polymers are simultaneously observed. The boundary between SAOS and LAOS is called a linear viscoelastic (LVE) region. We observed that the LVE of CPU1~3 was 5% through an amplitude sweep analysis at 180 °C, which was judged as the SRA limit temperature (Figure S3). Thus, the CPU1~3 films were subjected to a constant normal force of 5 N and a strain of 1% in a rheometer, and the relaxed stress G(t) was observed over heating time (t) and plotted with normalization. The initial stress on the CPU1 film was gradually relaxed (Figure 1b), and the rate of relaxation increased at high temperatures. This stress relaxation phenomenon is a characteristic feature of CANs because conventional thermosets do not show such relief, except for some creep. Thus, we attributed this stress relaxation to the transcarbamoylation (urethane exchange reactions) in CPU1. Under similar conditions, the relaxation behavior of CPU3 film was extremely similar to that of CPU1, but that of CPU2 was slightly different from these two (Figure S4). At 150 °C, the relaxation times (τ) for CPU1, CPU2, and CPU3, determined by 37% (1/e) of the original modulus according to the Maxwell model, were observed to be approximately 420, 800, and 290 s, respectively (Figure 1c) [32,47]. Notably, CPU2 contains phenyl groups from FCT-2, which may hinder the movement of polymer chains inside its network (Figure 1a). Therefore, it may take a long time for CPU2 to show stress relaxation. Interestingly, the branched isopropyl group in CPU3 appears to enhance chain motion and reduce relaxation time, and this becomes more pronounced at high temperatures.

Figure 1d shows that relaxation time plots (τ) for CPUs against the inverse temperatures (1/T) yield activation energies (E_a_), assuming Arrhenius behaviors. We assume that the stress relaxations in CPU films occur through transcarbamoylation, carried out with only 1 mol% of DBTDL catalyst. Considering previous studies, the transcarbamoylation E_a_ value showed various values between 80 and 180 depending on the reaction conditions. E_a_ values of CPU1, CPU2, and CPU3 are 150, 128, and 172, respectively, which is similar to the activation energy range observed in previous studies [48,49,50].

### 3.3. Thermal Conductivity Analysis of CPU–Ag Composites as Thermal Interfacial Material Applications

To test CPUs as a matrix polymer for TIM, CPU–Ag composites were prepared. As reported in our previous studies, Ag flake- and silver-nanoparticle-decorated multiwalled carbon nanotube (nAgMWNTs) combination is a filler with excellent thermal conductivity, which can greatly improve the thermal conductivity of polymer-based TIMs through well-designed thermal percolation pathways. The 30- and 50-vol% Ag flakes and nAgMWNTs were added at the same time during the ball milling synthesis of CPUs (Scheme 1b). When Ag flakes were added at a vol% exceeding 30 vol%, the resulting composite seemed too brittle and hard to fabricate into films, so we determined that the optimal amount of Ag flake was 30 vol%. Through this process, a CPU–thermal filler composite powder (CPU–Ag), containing dispersed Ag in CPU networks, was obtained. The CPU–Ags were also fabricated into a film through a process similar to that use for CPU films. The incorporation of Ag flakes into the CPU networks was confirmed through scanning electron microscopy (SEM) images and their EDS mapping (Figure S5).

In order to investigate the effect of Ag addition on the thermal and dynamic mechanical properties, we attempted several times to obtain stress relaxation curves for CPU–Ag composites with a rheometer under the same conditions as “undoped” CPUs. But unfortunately, we were unable to obtain the stress relaxation profiles sufficiently to analyze thermomechanical properties due to some instability (Figure S6). We suspect that Ag flakes may hinder the rheological measurements, but it does not mean that there are no exchange reactions between urethane bonds. We confirmed that the broken pieces of CPU–Ag composites can be reprocessed to new films (Figure S7), which indicates that they are, in fact, CANs. When a film of CPU1–Ag was cut into pieces and reprocessed using a hot press at 200 °C under 20 MPa for 2 h, the film was reformed. When analyzing DSC and TGA measurements, we observed an enhancement in the thermal properties of the CPU by Ag addition. The T_g_ measured by DSC was −4 °C before the addition of Ag, but it increased by approximately 20 °C after the incorporation of Ag (Figure S8). Additionally, TGA measurements showed a significantly higher T_d95%_ value following the addition of Ag; while the CPU1 decomposed at 181 °C, the CPUa–Ag decomposed at 282 °C, showing an approximate 100 °C increase in the T_d95%_ value.

**Figure 2:**
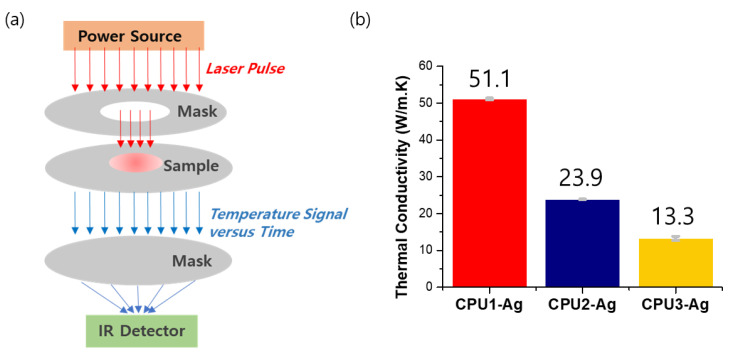
(**a**) Schematic diagram of laser flash analysis (LFA) process; (**b**) thermal conductivities of CPU–Ag TIMs, measured using LFA.

The thermal conductivities of CPU–Ag composites were evaluated using LFA (Figure 2). LFA is a technique used to explore the thermal diffusivity of a wide range of materials and perform thermal analysis. After subjecting one side of the sample to laser irradiation to induce heating, we measured the temperature rise in the sample on the opposite side to determine the time temperature reached its maximal saturation (Figure 2a). This information enabled us to calculate the thermal diffusivity (α) through the film, which could be correlated with specific heat (Cp) and density (ρ) values to determine the film’s thermal conductivity (κ). The measured ρ and Cp for CPU–Ag films in this study showed similar values, 4.1–4.7 g/cm^3^ and 0.41–0.55 J/g·K (Table S1). However, CPU1–Ag showed a high α (26.522 mm^2^/s) that was more than twice as high as those of other CPU–Ag composites (5.9–10.4 m^2^/s), as confirmed by LFA, which showed that CPU1 appeared to be the best matrix for TIMs (κ = 51.1 W/m·K) (Figure 2b). Although CPU3–Ag had the lowest κ of 13.3 W/m·K, it showed a higher thermal conductivity than commercially available TIMs (2–4 W/m·K) [51]. These results that show high thermal conductivities for CPU–Ag composites could support two hypotheses. First, during the polymerization that occurred in-situ during the ball milling process, the filler was effectively dispersed even in the solid phase (the polymer matrix). Second, many gecarbamate bonds in the network structure of the polyurethanes could effectively bind to silver flakes to produce thermal fillers having excellent percolation. The reason for the highest κ of CPU1–Ag achieved by the simple FCT-1 when compared with the phenyl CPU2–Ag and branched CPU3–Ag is not evident at this point. We suspected that CPU1–Ag, which has the simplest structure, has a more ordered or packed structure than other samples, leading to better thermal conductivity between chains. This hypothesis seems to be supported by the density data (Table S1), which shows that CPU1 has the highest density.

## 4. Conclusions

We successfully prepared novel network polyurethanes (CPUs) from biomass-derived furan material (BHMF) and CO_2_-fixated monomers (FCTs) via a facile ball milling synthesis then further processed them into composites using thermal conductive fillers for TIM applications. The chemical structure of FCTs affected the thermomechanical properties of CPUs; phenyl side groups appeared to hinder chain movements, resulting in a high value for T_g_. Furthermore, the rheological analysis of CPUs confirmed their characteristic CAN feature from transcarbamoylation, and the phenyl side groups seemed to result in slow relaxation times. In addition, the excellent thermal conductivities of the composites were demonstrated using LFA. CPU1–Ag showed a very high thermal conductivity of 51.1 W/m·K, when compared to the thermal conductivity value of 2–4 W/m·K of commercially available TIMs. Our approach showed successfully that network polymer composites containing CANs can be prepared from biomass-derived and CO_2_-fixated monomers using a mechanochemical method. We believe our approach could be utilized in the production of environmentally friendly and sustainable TIMs having high thermal conductivity for high-performance electronic devices.

## Data Availability

The data presented in this study are available on request from the corresponding authors.

## Supplementary Materials

The following supporting information can be downloaded at: https://www.mdpi.com/article/10.3390/polym16020177/s1, Table S1: The diffusivity, density, specific heat, and thermal conductivity of CPU–Ag composites, Figure S1: FT-IR spectra of CPU1~3 and starting materials, Figure S2: DSC and TGA curves of CPU1~3 powder, Figure S3: Storage modulus in the strain sweep of CPU1~3 at 180 °C, Figure S4: Comparison of stress relaxation profiles of CPU1, CPU2, and CPU3 at (a) 140 °C, (b) 150 °C, and (c) 160 °C. (d) Arrhenius plot of the measured relaxation times for CPU1, CPU2, and CPU3, Figure S5: SEM images of CPU1–Ag (a) and CPU2–Ag (b). SEM-EDS mapping of CPU1–Ag (c) and CPU2–Ag, Figure S6: (a) Stress relaxation analysis of CPU1–Ag at 140 °C, 150 °C, and 160 °C. (b) Stress relaxation analysis of CPU2–Ag at 140 °C, 150 °C, and 160 °C, Figure S7: Malleability test of CPU1–Ag, Figure S8: Comparisons of DSC (a) and TGA curves (b) of CPU1 and CPU1–Ag, Figure S9: ^1^H and ^13^C NMR spectra of FCT-1, Figure S10: ^1^H and ^13^C NMR spectra of FCT-2, Figure S11: ^1^H and ^13^C NMR spectra of FCT-3.
