# Supplementary material for: Biomass- and Carbon Dioxide-Derived Polyurethane Networks for Thermal Interface Material Applications
Source: Polymers (Basel). 2024 Jan 7;16(2):177. doi: 10.3390/polym16020177 (PMC10820237; doi:10.3390/polym16020177)
Supplement: Supplementary file 1 [file polymers-16-00177-s001.zip › polymers-2745203-supplementary.pdf]

Supplementary Materials

Sustainable Network Polyurethanes Using Carbon Dioxide and Biomass-Derived Alcohols as Thermal Interface Materials

Ji Won Jang[1†], Inhwhan Cha[1†], Junhyeon Choi[2†], Jungwoo Han[1], Joon Young Hwang[2], Seung Uk Son[1], Eun Joo Kang[2], and Changsik Song[1]**

[1]Department of Chemistry, Sungkyunkwan University, Suwon, 16419, Republic of Korea

[2]Department of Applied Chemistry, Kyung Hee University, Yongin, 17104, Republic of Korea

*E-mail: songcs@skku.edu ; ejkang24@khu.ac.kr.

Table of Contents

- ✓ **Table S1** The diffusivity, density, specific heat, and thermal conductivity of CPU-Ag composites. S2
- ✓ **Figure S1** FT-IR spectra of CPU1~3 and starting materials S3
- ✓ **Figure S2** DSC and TGA curves of CPU1~3 powder S4
- ✓ **Figure S3.** Storage modulus in the strain sweep of CPU1~3 at 180 °C. S5
- ✓ **Figure S4** Comparison of stress relaxation profiles of CPU1, CPU2, and CPU3 at (a) 140 °C, (b) 150 °C, and (c) 160 °C. (d) Arrhenius plot of the measured relaxation times for CPU1, CPU2, and CPU3. .... S6
- ✓ **Figure S5.** SEM images of CPU1-Ag (a) and CPU2-Ag (b). SEM-EDS mapping of CPU1-Ag (c) and CPU2-Ag (d). S7
- ✓ Figure S6. Stress relaxation analysis at 140 °C, 150 °C, 160 °C of CPU1-Ag (a) and CPU2-Ag (b). S8
- ✓ **Figure S7.** Malleability test of CPU1-Ag S9
- ✓ **Figure S8.** Comparisons of DSC (a) and TGA curves (b) of CPU1 and CPU1-Ag S10
- ✓ **Figure S9** ^{1}H and ^{13}C NMR spectra of FCT-1 .... S11
- ✓ **Figure S10** ^{1}H and ^{13}C NMR spectra of FCT-2 .... S12
- ✓ **Figure S11** ^{1}H and ^{13}C NMR spectra of FCT-3 .... S13

Table S1. The diffusivity, density, specific heat, and thermal conductivity of CPU-Ag composites.

Name	α[a] (mm^2/s)	ρ[b] (g/cm^3)	C_p[c] (J/g.K)	κ[d] (W/m.K)
**CPU1-Ag**	26.522	4.654	0.414	51.1
**CPU2-Ag**	10.382	4.216	0.546	23.9
**CPU3-Ag**	5.948	4.187	0.534	13.3

[a]Thermal diffusivity of composites was measured by LFA (LFA467). [b]Density was measured in 25 °C. [c]Specific heat capacity was measured by specific heat DSC in 25 °C. [d]Thermal conductivity of composites was calculated by using the equation: $\kappa = \alpha \times \rho \times C_p$

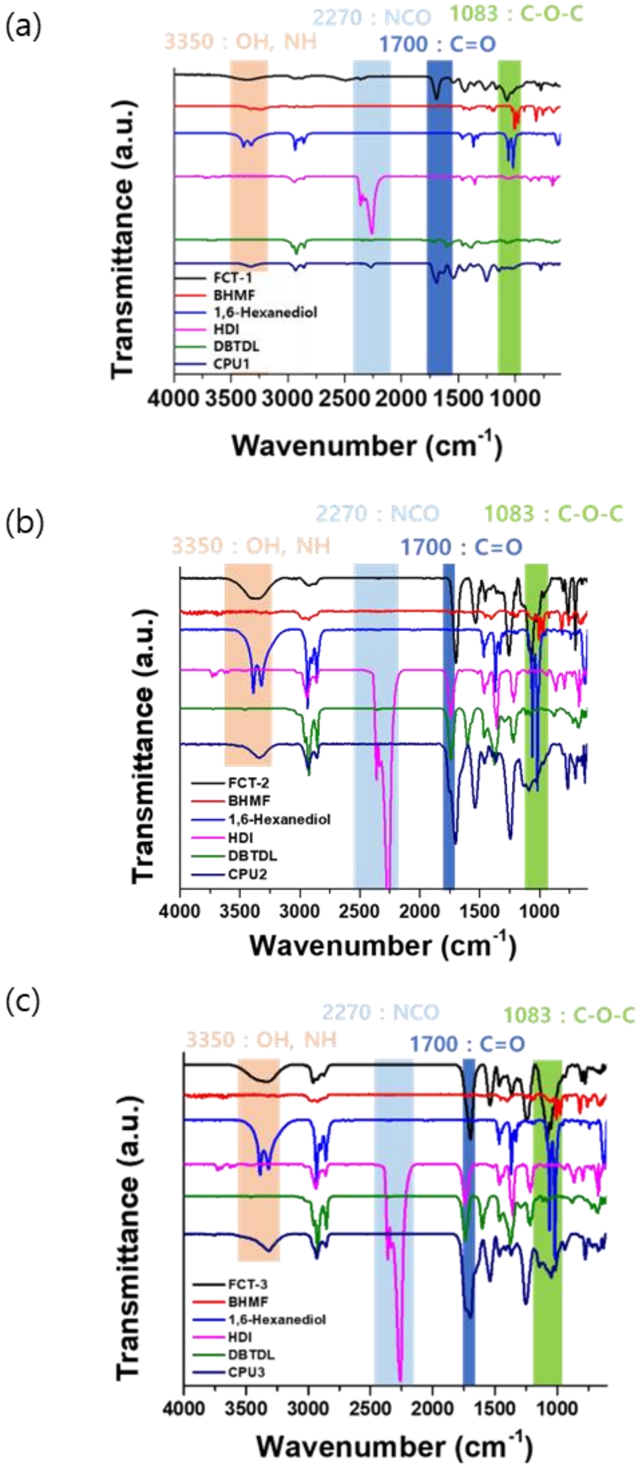

Figure S1. (FT-IR spectra of CPU1~3 and starting materials

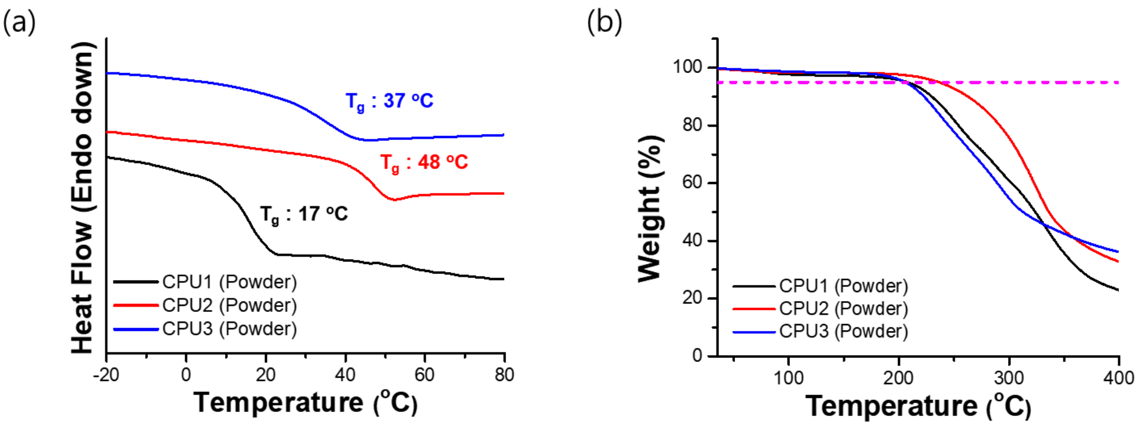

Figure S2. DSC and TGA curves of CPU1~3 powder

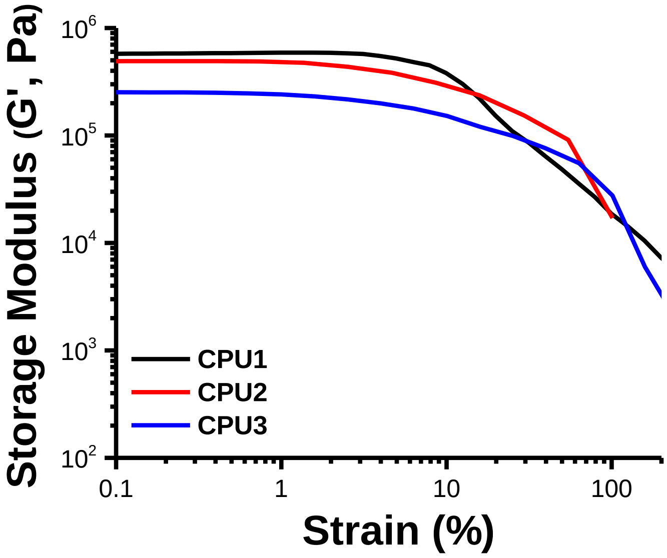

Figure S3. Storage modulus in the strain sweep of CPU1~3 at 180 °C.

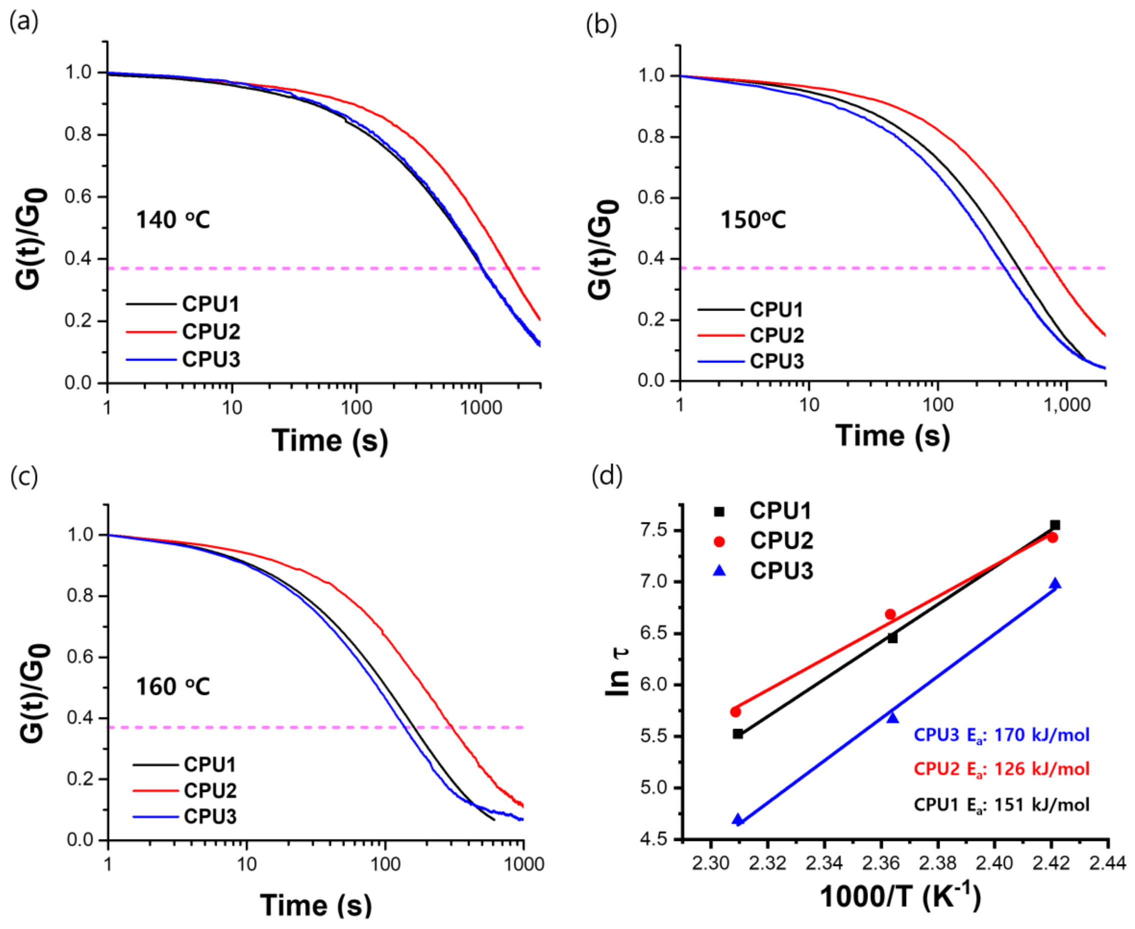

Figure S4. Comparison of stress relaxation analysis of CPU1, CPU2, and CPU3 at (a) 140 °C, (b) 150 °C, (c) 160 °C (d) Arrhenius plot of the measured relaxation times for CPU1, CPU2, and CPU3

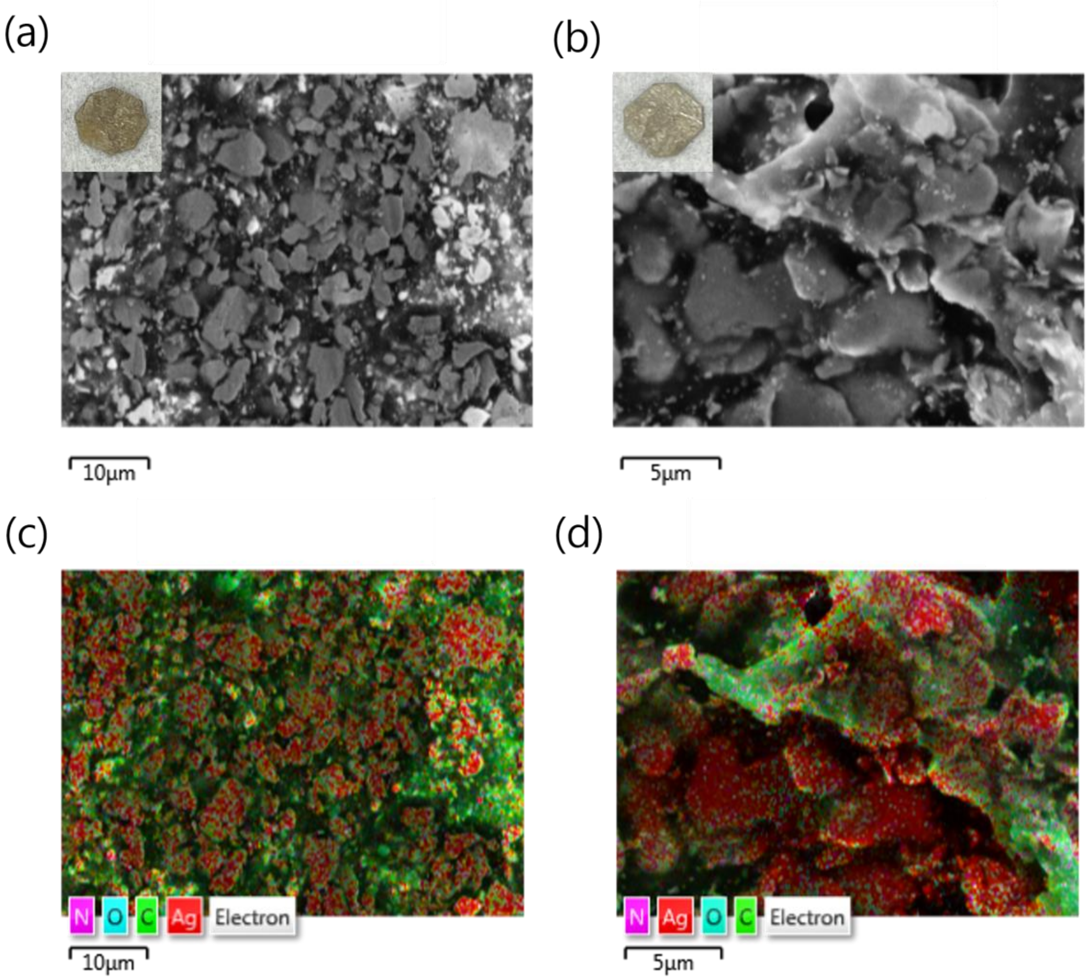

Figure S5. SEM images of CPU1-Ag (a) and CPU2-Ag (b). SEM-EDS mapping of CPU1-Ag (c) and CPU2-Ag (d)

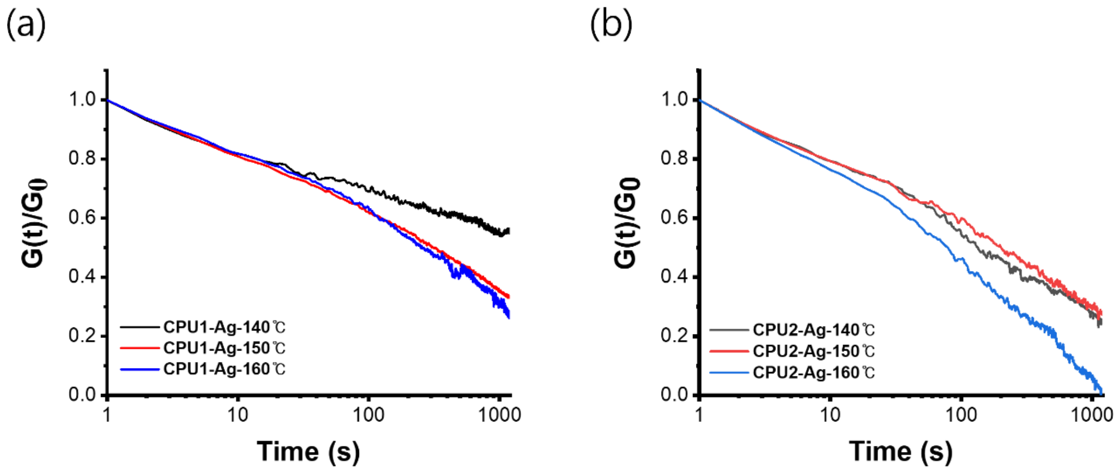

Figure S6. Stress relaxation analysis at 140 °C, 150 °C, 160 °C of CPU1-Ag (a) and CPU2-Ag (b).

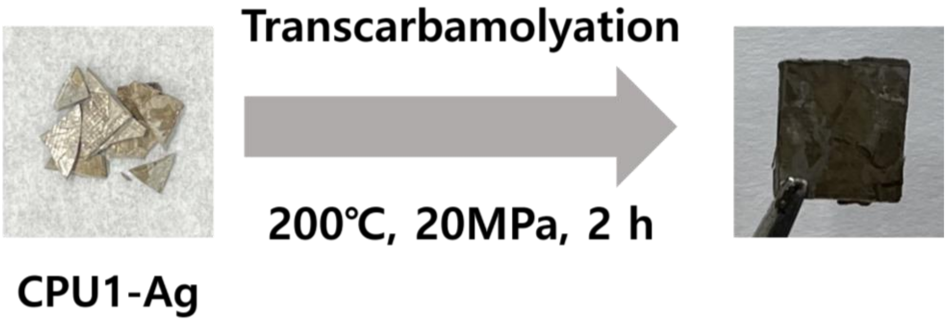

Figure S7. Malleability test of CPU1-Ag

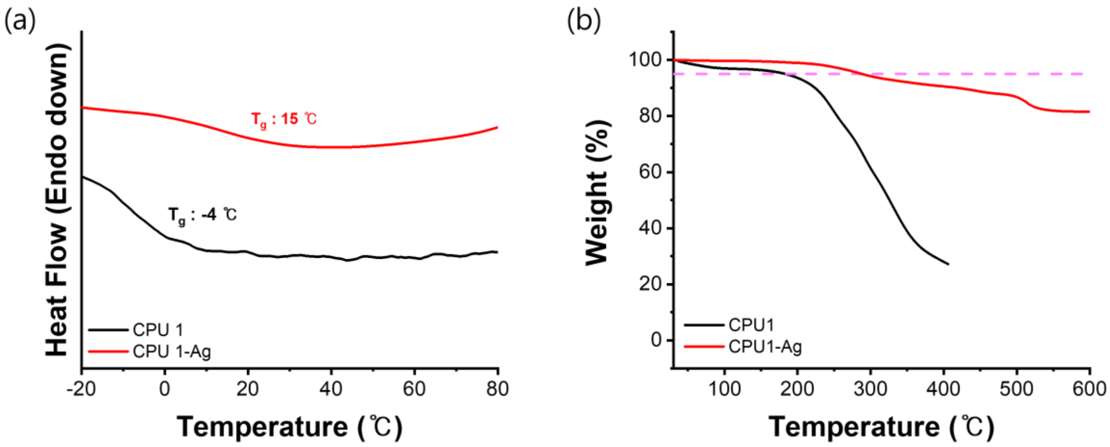

Figure S8. Comparisons of DSC (a) and TGA curves (b) of CPU1 and CPU1-Ag

(a)

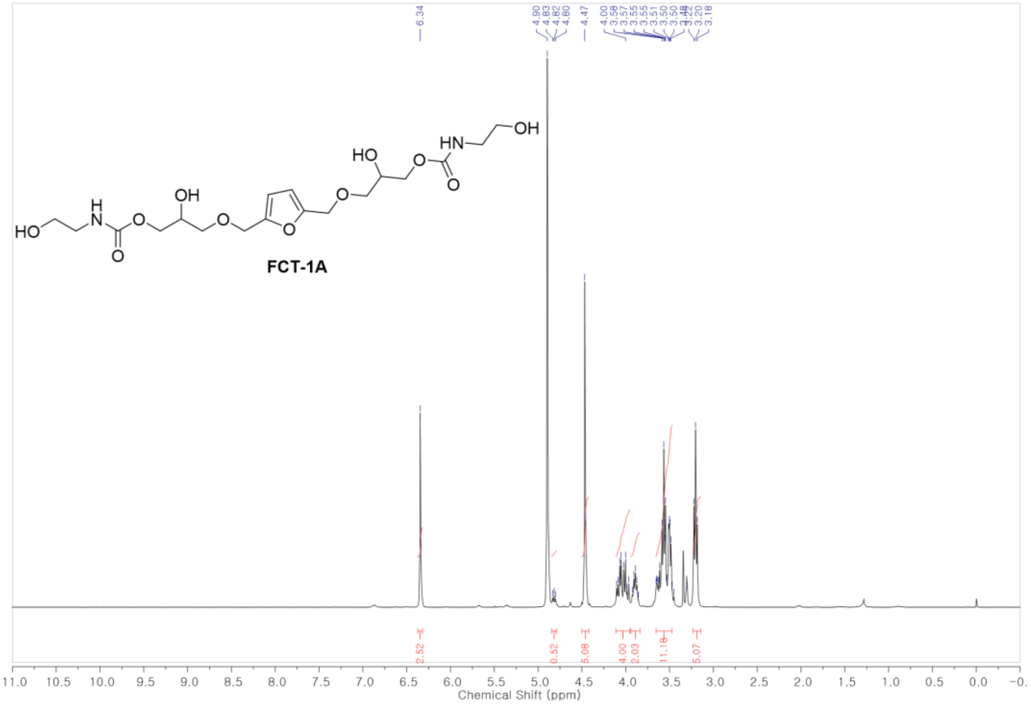

(b)

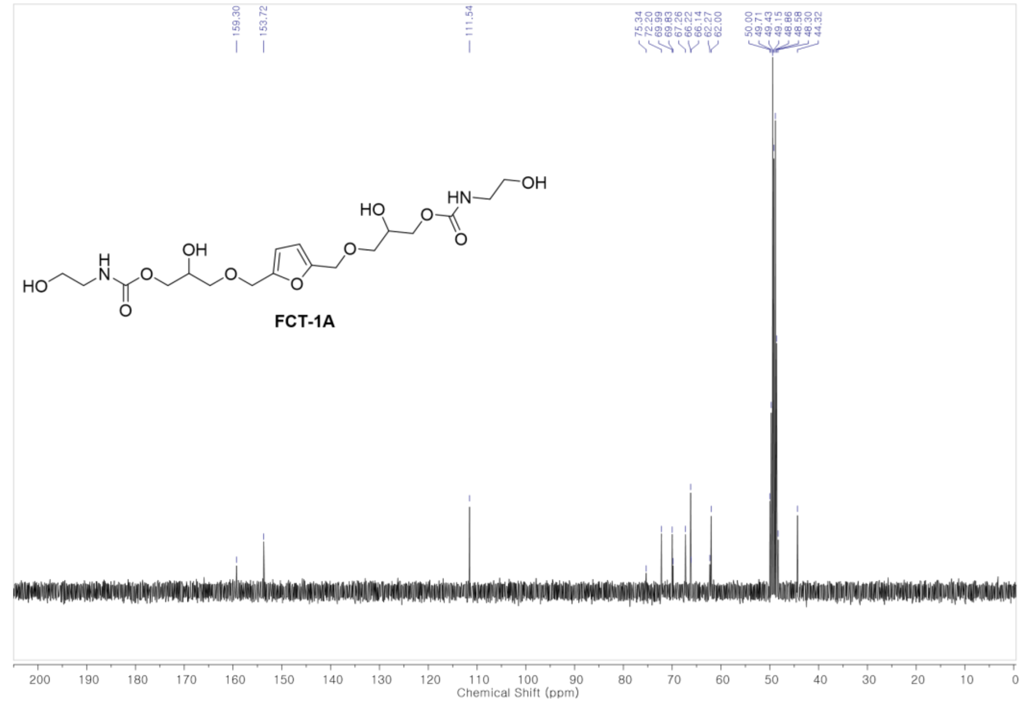

Figure S9. ^{1}H (300 MHz, CD_3OD) (a) and ^{13}C NMR spectra (85 MHz, CD_3OD) (b) of FCT-1.

(a)

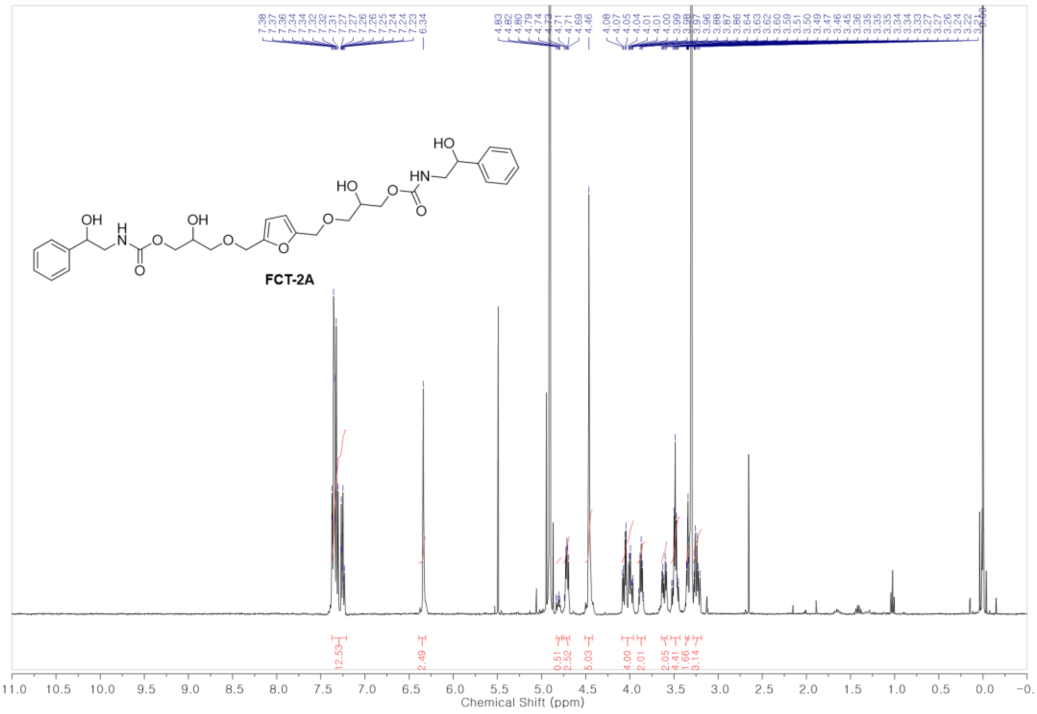

(b)

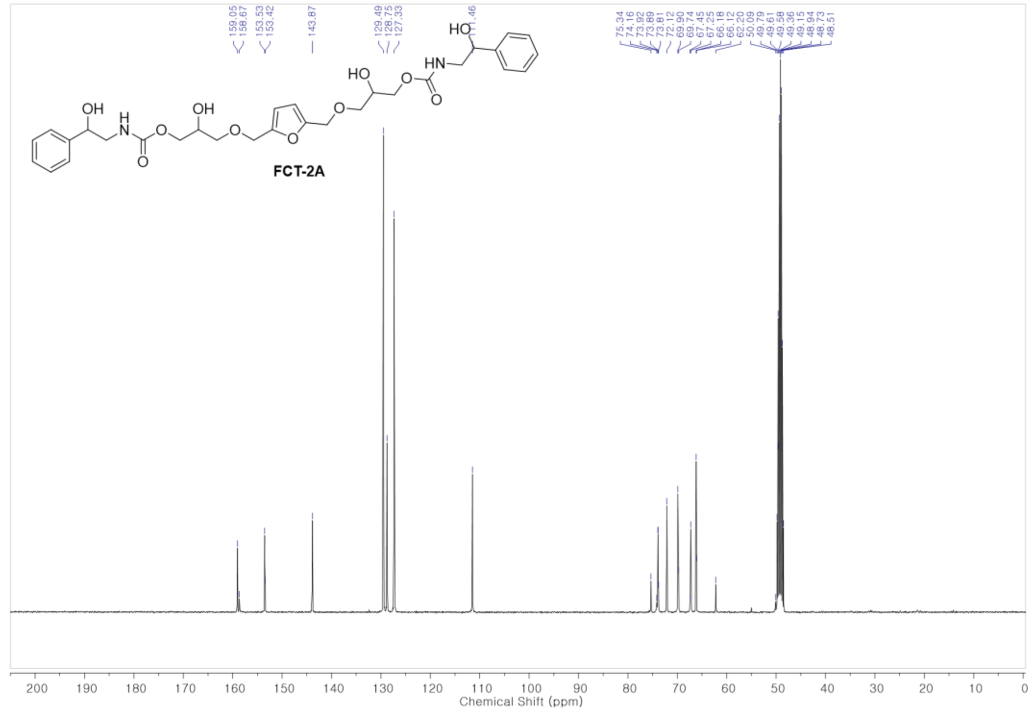

Figure S10. ^{1}H (300 MHz, CD_3OD) (a) and ^{13}C NMR spectra (85 MHz, CD_3OD) (b) of FCT-2.

(a)

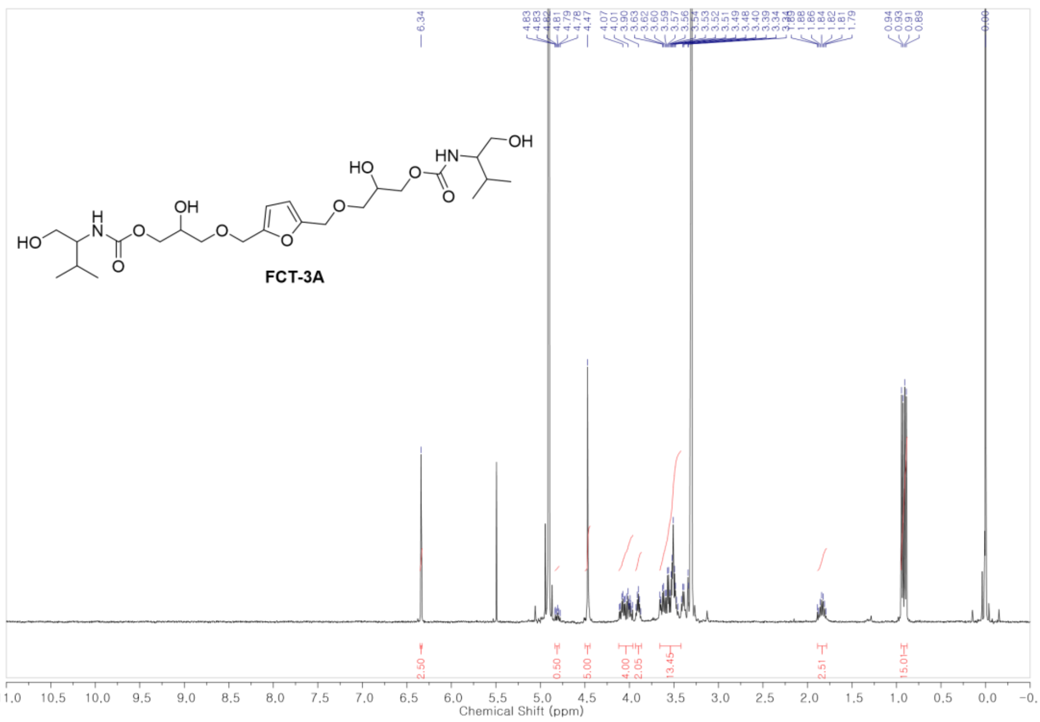

(b)

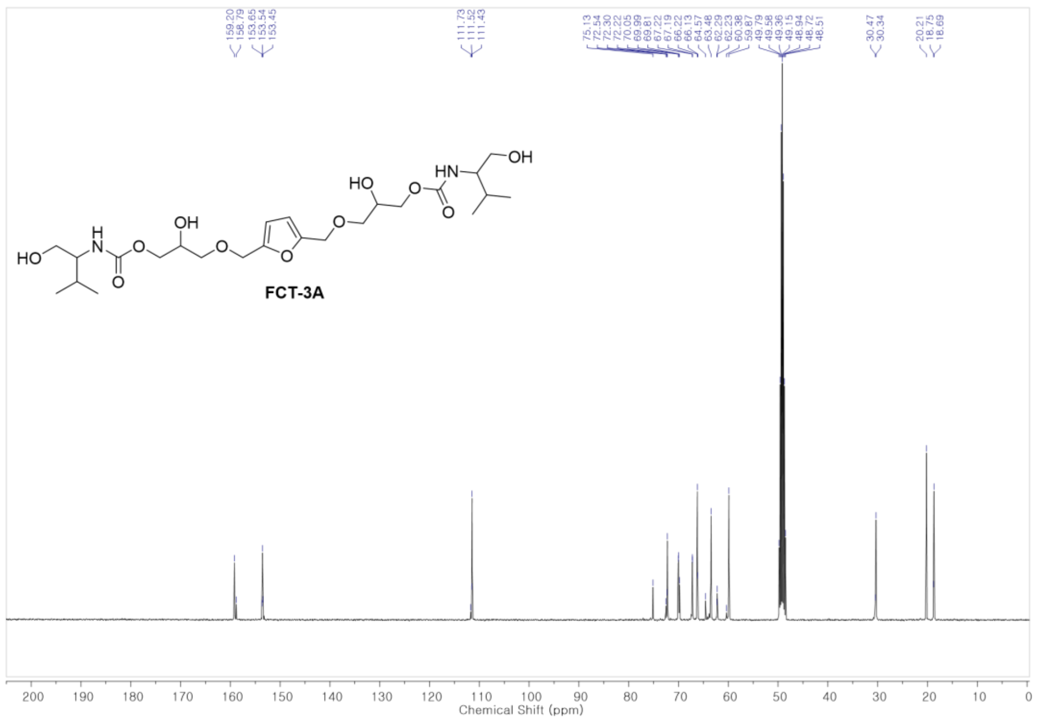

Figure S11. ^{1}H (300 MHz, CD_3OD) (a) and ^{13}C NMR spectra (85 MHz, CD_3OD) (b) of FCT-3.
